# A fatal pseudo-tumour: disseminated basidiobolomycosis

**DOI:** 10.1186/1471-2334-6-140

**Published:** 2006-09-15

**Authors:** Guido EL van den Berk, L Arnold Noorduyn, Ruud J van Ketel, Jeannouel van Leeuwen, Willem A Bemelman, Jan M Prins

**Affiliations:** 1 Department of Internal Medicine, Division of Infectious Diseases, Tropical Medicine and AIDS, Academic Medical Center, Amsterdam, the Netherlands; 2 Department of Pathology, Academic Medical Center, Amsterdam, the Netherlands; 3 Department of Medical Microbiology, Academic Medical Center, Amsterdam, the Netherlands; 4 Department of Surgery, Hospital Curaçao, Curaçao, Dutch Antilles; 5 Department of Surgery, Academic Medical Center, Amsterdam, the Netherlands

## Abstract

**Background:**

Basidiobolomycosis is a rare disease caused by the fungus *Basidiobolus ranarum*, member of the class *Zygomycetes*, order *Entomophthorales*, found worldwide. Usually basidiobolomycosis is a subcutaneous infection but rarely gastrointestinal manifestations have been described; 13 adults and 10 children and a few retroperitoneal or pulmonary cases. In gastrointestinal basidiobolomycosis the colon is most frequently involved, usually presenting with subacute mild abdominal pain. In contrast to children only very few described adult patients had hepatic masses. Definitive diagnosis requires culture, serological testing can be helpful. The fungal morphology and the Splendore-Hoeppli phenomenon are characteristic histological features. There are no prominent risk factors. Usually surgery and prolonged antifungal therapy are required.

**Case presentation:**

A 61 year old man presented with progressive left abdominal pain and constipation since a few months. Colonoscopy showed an obstructing tumour in the descending colon, and a hemicolectomy was performed. Histology showed inflammation, possibly caused by a fungal or parasitic infection, without definite identification of an organism. A few weeks postoperatively a CT scan made because of abdominal discomfort, revealed a livermass (6 cm). Treatment with metronidazole, directed against an amoebic liver abscess, was unsuccessful. He developed a marked eosinophilia (27.7%). A liver biopsy was performed and the patient was referred to a university hospital.

A repeated CT scan showed a livermass of 9 cm diameter. Review of colon and liver biopsy samples showed extensive necrosis and histiocytes, multinucleated giant cells and numerous eosinophils. Grocott stained sections contained unusually large hyphae surrounded by strongly eosinophilic material in haematoxylin and eosin stained sections (Splendore-Hoeppli phenomenon). A presumptive diagnosis of *Basidiobolus *spp. infection was made and treated with amphotericin B (Itraconazol contra-indicated because of renal insufficiency). A few days later the patient died of a septic shock. After autopsy *Basidiobolus ranarum *was cultured from liver, gallbladder and colon.

**Conclusion:**

Our patient died of gastrointestinal basidiobolomycosis with an obstructing colon tumour and a large hepatic mass. This was a rare presentation of basidiobolomycosis and the second fatal case described worldwide.

## Background

Basidiobolomycosis is a rare disease caused by the fungus *Basidiobolus ranarum*, an environmental saprophyte, member of the class *Zygomycetes*, order *Entomophthorales*, found worldwide [1]. Usually basidiobolomycosis is a subcutaneous infection that is transmitted through traumatic inoculation [1]. Gastrointestinal basidiobolomycosis is rare with only 13 cases reported worldwide in adults [2,3] and 10 in children. [4,5]. Only a few cases of retroperitoneal [6-8] or pulmonary [9] basidiobolomycosis have been reported. In gastrointestinal basidiobolomycosis the colon is the most frequently involved part of the gastrointestinal tract, and patients usually present with mild abdominal pain with a subacute onset, eosinophilia, and on histopatologic examination inflammatory changes with many eosinophils [2]. In contrast to pediatric patients only very few of the reported adult patients also had a hepatic mass [4,5,10]. Definitive diagnosis requires culture of the organism, serological testing via an immunodiffusion method can be helpful [1]. Because a fungal infection is not always suspected, in a number of patients the diagnosis must be made on histology alone. The fungal morphology and the Splendore-Hoeppli phenomenon, although not entirely specific, are characteristic histological features.

There are no prominent risk factors for this disease [2]. For treatment surgery is usually required, followed by prolonged antifungal therapy [1,2]. The preferred drug is itraconazole [2].

## Case presentation

A 61 year old man presented with progressive lower left abdominal pain and constipation since a few months. Colonoscopy showed a large obstructing tumour in the descending colon, and a hemicolectomy was performed. Histology showed an inflammatory reaction, possibly caused by a fungal or parasitic infection, but no definite identification of an organism was made. Postoperatively his complaints disappeared, but after a few weeks he developed abdominal discomfort in his right upper abdomen, and six weeks postoperatively a CT scan revealed a large mass with a diameter of 6 cm central in the right liver lobe. Treatment with metronidazole, directed against an amoebic liver abscess, was unsuccessful. A subsequent four-week course with fluconazole resulted in a small decrease of the liver abscess, without clinical improvement. He developed a marked eosinophilia (27.7%). A liver biopsy was performed and the patient was referred to a university hospital.

**Figure 1:**
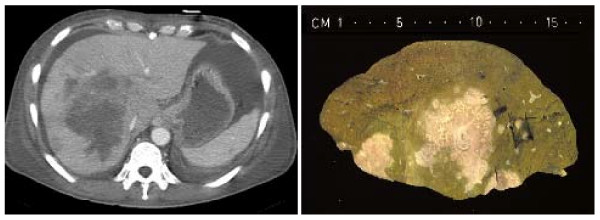
A. CT scan of the abdomen showing a large mass central in the liver, with extension to the right liver lobe. B. Postmortem aspect of the liver, with extensive fungal infection.

**Figure 2:**
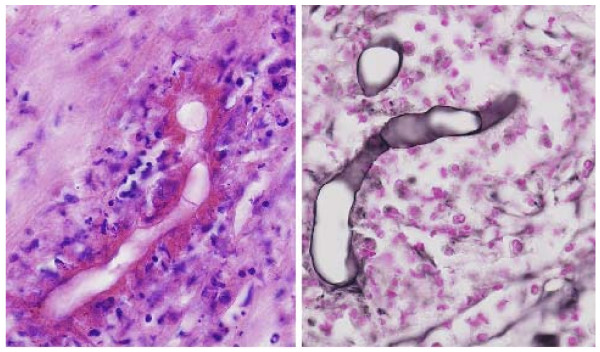
Liver biopsy, showing large hyphae surrounded by strongly eosinophilic material and an inflammatory cell infiltrate containing histiocytes, multinucleated giant cells and numerous eosinophils. (a) H&E stained (b) Grocott stained sections.

A repeated CT scan showed a large mass with a diameter of 9 cm central in the liver, with extension to the right liver lobe (figure 1A). Review of the slides from the colonic mass and the liver biopsy showed similar features, with extensive necrosis and a mixed inflammatory cell infiltrate containing histiocytes, multinucleated giant cells and numerous eosinophils. In Grocott stained sections, many unusually large hyphae could be recognized which were surrounded by strongly eosinophilic material in haematoxylin and eosin stained sections (Splendore-Hoeppli phenomenon) (figure 2).

On the basis of this morphology, a presumptive diagnosis of infection with *Basidiobolus *spp. was made. A percutaneous cholangiodrain was placed to treat the cholestasis caused by the hepatic mass. A few days later, the patient developed a septic shock, probably of hepatic origin. Blood cultures yielded *Escherichia coli *and *Clostridium perfringens*. The patient was treated with broad-spectrum antibiotics. The presumed basidiobolomycosis was treated with amphotericin B intravenously, because the preferred therapy, intravenous itraconazole, was contra-indicated because of severe renal insufficiency. A few days after initiation of the antifungal therapy the patient died of multiple organ failure. Postmortem autopsy showed signs of extensive fungal infection of the liver (figure 1B), gallbladder and sigmoid colon. Culture of liver, gallbladder and sigmoid colon yielded *Basidiobolus ranarum*.

## Conclusion

Our patient died of gastrointestinal basidiobolomycosis with an obstructing colon tumour and a large hepatic mass. There was difficulty in reaching the diagnosis. The case provides a teaching point, because although gastrointestinal basidiobolomycosis is a rare disease, the clinical presentation of our patient was characteristic for this disease. The prognosis of gastrointestinal basidiobolomycosis is usually favourable, our patient is the second in whom the outcome was fatal [9]. Better familiarity with this condition may prevent a fatal outcome like in our patient

## Competing interests

The author(s) declare that they have no competing interests.

## Authors' contributions

All authors read and approved the final manuscript

1) GEL participated in the clinical care of the patient and the writing of the case report. Furthermore, he has seen and approved the final version.

2) LA participated in reviewing the samples of colon and liver of the patient described and in the writing of the case report. Furthermore, he has seen and approved the final version.

3) RJ participated in the clinical care of the patient described, in reviewing the samples from colon and liver and in the writing of the case report. Furthermore, he has seen and approved the final version.

4) J participated in the clinical care of the patient and in the writing of the case report. Furthermore, he has seen and approved the final version.

5) WA participated in the clinical care of the patient and in the writing of the case report and I have seen and approved the final version. Furthermore, he has seen and approved the final version.

6) JM participated in the clinical care of the patient and in the writing of the case report. Furthermore, he has seen and approved the final version.

## Pre-publication history

The pre-publication history for this paper can be accessed here:


